# Brain state-dependent repetitive transcranial magnetic stimulation for motor stroke rehabilitation: a proof of concept randomized controlled trial

**DOI:** 10.3389/fneur.2024.1427198

**Published:** 2024-08-26

**Authors:** Wala Mahmoud, David Baur, Brigitte Zrenner, Arianna Brancaccio, Paolo Belardinelli, Ander Ramos-Murguialday, Christoph Zrenner, Ulf Ziemann

**Affiliations:** ^1^Institute for Clinical Psychology and Behavioral Neurobiology, University of Tübingen, Tübingen, Germany; ^2^Department of Neurology and Stroke, University of Tübingen, Tübingen, Germany; ^3^Hertie Institute for Clinical Brain Research, University of Tübingen, Tübingen, Germany; ^4^Temerty Centre for Therapeutic Brain Intervention, Centre for Addiction and Mental Health, Toronto, ON, Canada; ^5^Department of Psychiatry, University of Toronto, Toronto, ON, Canada; ^6^Center for Mind/Brain Sciences—CIMeC, University of Trento, Rovereto, Italy; ^7^Tecnalia, Basque Research and Technology Alliance, San Sebastián, Spain; ^8^Athenea Neuroclinics, San Sebastián, Spain; ^9^Institute for Biomedical Engineering, University of Toronto, Toronto, ON, Canada

**Keywords:** brain state-dependent stimulation, sensorimotor μ-oscillation, rTMS, motor stroke rehabilitation, spasticity

## Abstract

**Background:**

In healthy subjects, repetitive transcranial magnetic stimulation (rTMS) targeting the primary motor cortex (M1) demonstrated plasticity effects contingent on electroencephalography (EEG)-derived excitability states, defined by the phase of the ongoing sensorimotor μ-oscillation. The therapeutic potential of brain state-dependent rTMS in the rehabilitation of upper limb motor impairment post-stroke remains unexplored.

**Objective:**

Proof-of-concept trial to assess the efficacy of rTMS, synchronized to the sensorimotor μ-oscillation, in improving motor impairment and reducing upper-limb spasticity in stroke patients.

**Methods:**

We conducted a parallel group, randomized double-blind controlled trial in 30 chronic stroke patients (clinical trial registration number: NCT05005780). The experimental intervention group received EEG-triggered rTMS of the ipsilesional M1 [1,200 pulses; 0.33 Hz; 100% of the resting motor threshold (RMT)], while the control group received low-frequency rTMS of the contralesional motor cortex (1,200 pulses; 1 Hz, 115% RMT), i.e., an established treatment protocol. Both groups received 12 rTMS sessions (20 min, 3× per week, 4 weeks) followed by 50 min of physiotherapy. The primary outcome measure was the change in upper-extremity Fugl-Meyer assessment (FMA-UE) scores between baseline, immediately post-treatment and 3 months’ follow-up.

**Results:**

Both groups showed significant improvement in the primary outcome measure (FMA-UE) and the secondary outcome measures. This included the reduction in spasticity, measured objectively using the hand-held dynamometer, and enhanced motor function as measured by the Wolf Motor Function Test (WMFT). There were no significant differences between the groups in any of the outcome measures.

**Conclusion:**

The application of brain state-dependent rTMS for rehabilitation in chronic stroke patients is feasible. This pilot study demonstrated that the brain oscillation-synchronized rTMS protocol produced beneficial effects on motor impairment, motor function and spasticity that were comparable to those observed with an established therapeutic rTMS protocol.

**Clinical Trial Registration:**

ClinicalTrials.gov, identifier [NCT05005780].

## 1 Introduction

In recent years, repetitive transcranial magnetic stimulation (rTMS) has emerged as a safe and non-invasive neuromodulatory intervention for enhancing functional recovery after stroke. One fundamental principle of stroke rehabilitation employing rTMS is to enhance the excitability of the ipsilesional cortex. Two distinct stimulation protocols have been employed for that purpose: (1) high-frequency excitatory rTMS of the ipsilesional hemisphere, with the aim of direct enhancement of the corticospinal output; (2) low-frequency inhibitory rTMS of the contralesional hemisphere to restore excitability balance between the two cortical hemispheres (1–3).

There have been some promising findings for the application of low-frequency 1 Hz rTMS to the contralesional hemisphere for functional recovery (4–6) and reduction of spasticity (7). However, the results of various studies utilizing different rTMS protocols for stroke rehabilitation have not been consistent (5, 8). The effects, when found, are small and quite variable (9, 10), indicating that optimal stimulation protocols and parameters are not yet determined. The conventional “one-size-fits-all” approach to rTMS therapy has been criticized for neglecting individual patient characteristics. Thus, a shift towards individualized and patient-tailored rTMS therapies is advocated as a solution to reduce heterogeneity and to increase the overall therapeutic efficacy (11, 12).

Until recently, clinical research involving rTMS has employed an open-loop stimulation approach, disregarding the brain’s instantaneous state at the time of stimulation. The oscillatory activity of the neural networks represents rhythmic fluctuations in excitability (13–15), and significantly modulates the network’s response to various inputs (16–20). Therefore, taking into account the ongoing oscillatory brain state represents a compelling prospect for optimization of therapeutic brain stimulation.

Several investigations that explored EEG phase-dependent responses to TMS of the human primary motor cortex (M1) revealed that the trough/early rising phase of the endogenous sensorimotor μ-rhythm corresponds to a high-excitability state of corticospinal neurons, as indicated by motor evoked potentials (MEPs) of a larger amplitude compared to the peak of the μ-rhythm (21–24). Importantly, these alternating excitability states are decisive for the induction of plasticity: consistent triggering of rTMS during the high-excitability state led to long-term potentiation (LTP)-like effects in corticospinal excitability. In contrast, no changes were observed when stimuli were uncoupled from sensorimotor μ-phase in otherwise identical stimulation protocols (23, 25–27). In particular, the synchronization of TMS bursts with the trough of the μ-oscillation demonstrated efficacy in LTP-like plasticity induction in healthy subjects when presented in high-gamma frequencies (e.g., 100 Hz) (28).

Brain state-dependent TMS has predominantly been explored in healthy subjects. The method relies on autoregressive forward prediction to estimate future instantaneous oscillatory phases (23, 29), requiring consistent and predictable phase progression over time. In stroke, the disruption of brain networks can undermine the integrity of the μ-oscillation (30), which makes reliable phase targeting challenging. Nevertheless, Hussain et al. (31) have recently demonstrated in three chronic stroke patients that accurate targeting of the ipsilesional sensorimotor μ-rhythm is feasible.

Collectively, these findings suggest that real-time information about instantaneous brain states can be utilized to control the efficacy of plasticity in humans, potentially optimizing stimulation protocols to maximize the benefits of therapeutic interventions using rTMS in rehabilitation after stroke. The objective of this study was to investigate the feasibility and therapeutic efficacy of μ-phase-synchronized rTMS over M1 in improving motor disability of the upper limb in chronic stroke patients.

The application of rTMS for neurorehabilitation after stroke extends beyond treating motor disability to include the reduction of spasticity. Spasticity is defined as a “velocity-dependent increase in tonic stretch reflexes (muscle tone) with exaggerated tendon jerks, resulting from hyperexcitability of the stretch reflex” (32). The excitability of the monosynaptic Ia afferent-motoneuron (MN) pathway, which underlies the stretch reflex is regulated by intricate spinal circuitries, which are in turn modulated by supraspinal pathways descending from cortical and brainstem structures (33).

There is increasing evidence indicating that rTMS, particularly 1 Hz stimulation to the contralesional M1, effectively reduces spasticity (7). Although the precise mechanism by which rTMS might affect spasticity remains unclear, it is plausible that rTMS modulates the activity of spinal circuitry involved in spasticity by altering the excitability of cortical—or subcortical—centers projecting to this circuitry (34–36). We hypothesized that optimizing rTMS stimulation protocols through individualized brain state-dependent stimulation could enhance the effects not only on motor impairment and function but also on reducing spasticity. Therefore, as a secondary outcome measure in this feasibility trial, we aimed to assess the effects of rTMS on spasticity.

In summary, this study was designed to examine the feasibility and therapeutic efficacy of μ-phase-synchronized rTMS over M1 in improving motor disability of the upper limb in chronic stroke patients. A secondary outcome measure was to examine the effect on spasticity and the underlying mechanism. In this proof-of-concept trial, we compared the effects of μ-oscillation-triggered rTMS of the ipsilesional M1 with an established protocol of low-frequency rTMS over the contralesional M1.

## 2 Materials and methods

### 2.1 Trial design

This single-center parallel-group randomized double-blind controlled proof-of-concept trial compared brain-oscillation-triggered rTMS, synchronized with the trough of the EEG sensorimotor μ-rhythm of the ipsilesional hemisphere (referred to as intervention), with the established protocol of 1 Hz rTMS of the contralesional M1 (referred to as control). Both stimulation protocols were administered immediately prior to upper limb physiotherapy. Blinding procedures were implemented to ensure that participants and study staff, except for the personnel delivering the rTMS treatment, were unaware of group assignment.

The trial was conducted at Tübingen University Hospital-Department of Neurology & Stroke. All patients provided informed consent prior to participating in the trial (ethics committee approval No: 530/2019BO1). The study adhered to the principles of the declaration of Helsinki and was registered in a publicly accessible clinical trials registry (ClinicalTrials.gov, identifier: NCT05005780).

### 2.2 Subjects

**Figure 1:**
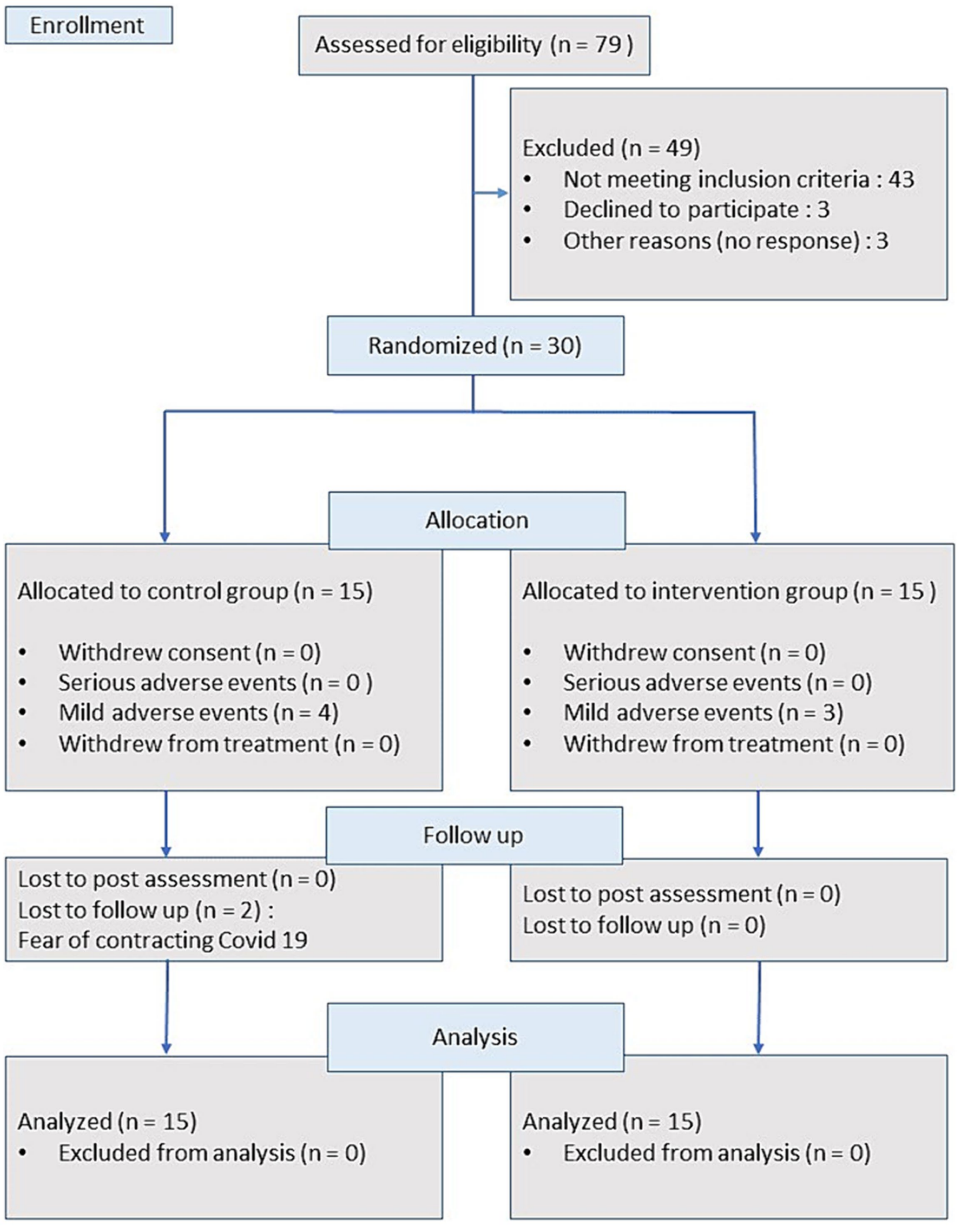
Flow chart of study participants.

We recruited 30 patients (23 males, 7 females) with upper limb hemiparesis following ischemic (*n* = 22) or hemorrhagic stroke (*n* = 8). The patients had an average age of 57.9 ± 8.0 (range: 39–73) years. The average time since stroke was 51 ± 47 months (range: 9 months—27.9 years). A total number of 79 subjects were screened for inclusion. Forty-nine of the screened subjects were excluded (43 did not meet the recruitment criteria, and 6 did not participate for other reasons). Figure 1 shows the participant flow chart, while subjects’ characteristics are found in Table 1.

**Table 1:** Characteristics of study participants.

ID	Age	Sex	Time since stroke (m)	Affected side	Ischemic /hemorrhagic	Location	FMA-UE/66	RMT (%)	MAS wrist	MAS total/70	Visual analog scale (%)	MAL (%)	Disability rating scale/24
1	70	F	40	R	Ischemic	No MRI	10	65	1	6.5	20	0	8
4	62	M	16	R	Hemorrhagic	Subcortical	22	80	1+	6.5	15	0	13
5	50	F	97	L	Ischemic	Cortical + subcortical	49	56	1+	9	50	39	11
7	69	M	120	R	Ischemic	Cortical + subcortical	39	59	1+	14	60	5	20
9	54	M	179	L	Ischemic	Cortical + subcortical	58	79	1	4	40	35	8
14	53	M	45	L	Ischemic	Subcortical	31	87	1	11	50	10	10
18	70	M	36	L	Ischemic	Cortical + subcortical	57	46	0	6	40	52	5
23	54	M	47	R	Hemorrhagic	No MRI	28	56	2	18.5	60	43	7
24	67	M	50	L	Ischemic	Subcortical	32	85	3	16.5	100	26	16
25	60	M	335	R	Ischemic	Subcortical	44	79	1+	10	30	28	7
32	39	M	12	R	Ischemic	Subcortical	38	58	1+	14.5	33	26	17
35	53	M	17	L	Ischemic	Subcortical	43	58	0	5.5	0	9	10
38	67	M	9	R	Ischemic	Subcortical	56	66	1	4	20	32	9
39	49	M	73	R	Ischemic	No MRI	38	72	3	17	75	14	4
41	50	F	14	L	Ischemic	No MRI	14	70	1	5	60	3	13
44	73	M	71	L	Ischemic	Subcortical	42	65	1+	9	30	21	11
45	61	M	66	R	Ischemic	Cortical + subcortical	30	69	1	12.5	50	7	6
47	53	F	33	R	Hemorrhagic	Cortical + subcortical	41	82	0	8.5	70	16	12
49	49	M	9	R	Ischemic	Cortical	38	56	0	17	75	14	4
55	58	M	27	R	Ischemic	Subcortical	56	59	0	0	0	30	7
60	61	M	35	L	Ischemic	Subcortical	41	54	1	8.5	40	20	7
61	66	M	36	R	Ischemic	Subcortical	34	42	1	10	55	18	12
62	55	M	15	L	Hemorrhagic	Subcortical	30	52	1+	6	30	23	4
65	52	M	29	L	Ischemic	No MRI	45	56	1	9	50	16	16
73	60	M	24	L	Hemorrhagic	No MRI	59	67	0	4	60	41	7
74	68	M	126	L	Hemorrhagic	Cortical + subcortical	19	76	1+	12	90	0	11
76	44	F	12	R	Ischemic	Cortical + subcortical	59	49	0	6.5	0	69	4
77	59	F	29	R	Hemorrhagic	Cortical + subcortical	21	60	2	15.5	30	0	20
78	58	M	22	L	Ischemic	Subcortical	23	84	1+	10	40	0	8
79	53	F	178	L	Hemorrhagic	Cortical + subcortical	17	59	1	12	80	7	13

The clinical scores represent those acquired at baseline. FMA-UE, Fugl-Meyer Assessment-Upper Extremity; RMT, resting motor threshold, expressed as a percentage of maximum stimulator output on the lesioned side; MAS, modified Ashworth scale; MAL (%), motor activity log, a structured interview which examines the patient’s use of the affected upper limb in comparison to the time before the injury.

The inclusion criteria were as follows: (1) minimum 6 months since stroke onset; (2) age 18–85 years and able to provide informed consent; (3) presence of ipsilesional MEPs; (4) resting motor threshold (RMT) of the contralesional M1 <80% of maximum stimulator output (MSO); (5) ability to understand and willingness to follow the Fugl-Meyer Assessment-Upper Extremity (FMA-UE) instructions, FMA-UE score ≤60.

Following safety and ethics guidelines for TMS in clinical practice and research (37), participants were excluded if they (1) had a seizure disorder history; (2) were taking pro-convulsive medication; (3) were taking muscle-relaxing medication (e.g., baclofen, tolperison, cannabis); (4) had a cardiac pacemaker, implanted medication pump, or an intracranial implant; (5) received a botulinum toxin injection in their affected upper limb <3 months before inclusion; (6) had a wrist joint contracture hindering spasticity measurement.

#### 2.2.1 Randomization

Patients were randomized into two groups of 15 patients each using a stratified block randomization method (38) with randomly selected block sizes of 4 or 6 patients. Stratification, based on FMA-UE scores, categorized patients into two strata: scores 0–30 and scores 31–60, ensuring a balanced distribution at baseline. The randomized list was generated using MATLAB.

### 2.3 Study overview

Patients underwent four evaluation sessions. The first session (pre-assessment) involved examination of eligibility based on inclusion and exclusion criteria, followed by baseline assessment, immediate post-therapy assessment, and a three-month follow-up. The study’s outcome measures were evaluated during all three assessment sessions. Adverse events were systematically monitored through inquiries after each rTMS session, addressing effects or complaints from the current or previous sessions.

**Figure 2:**
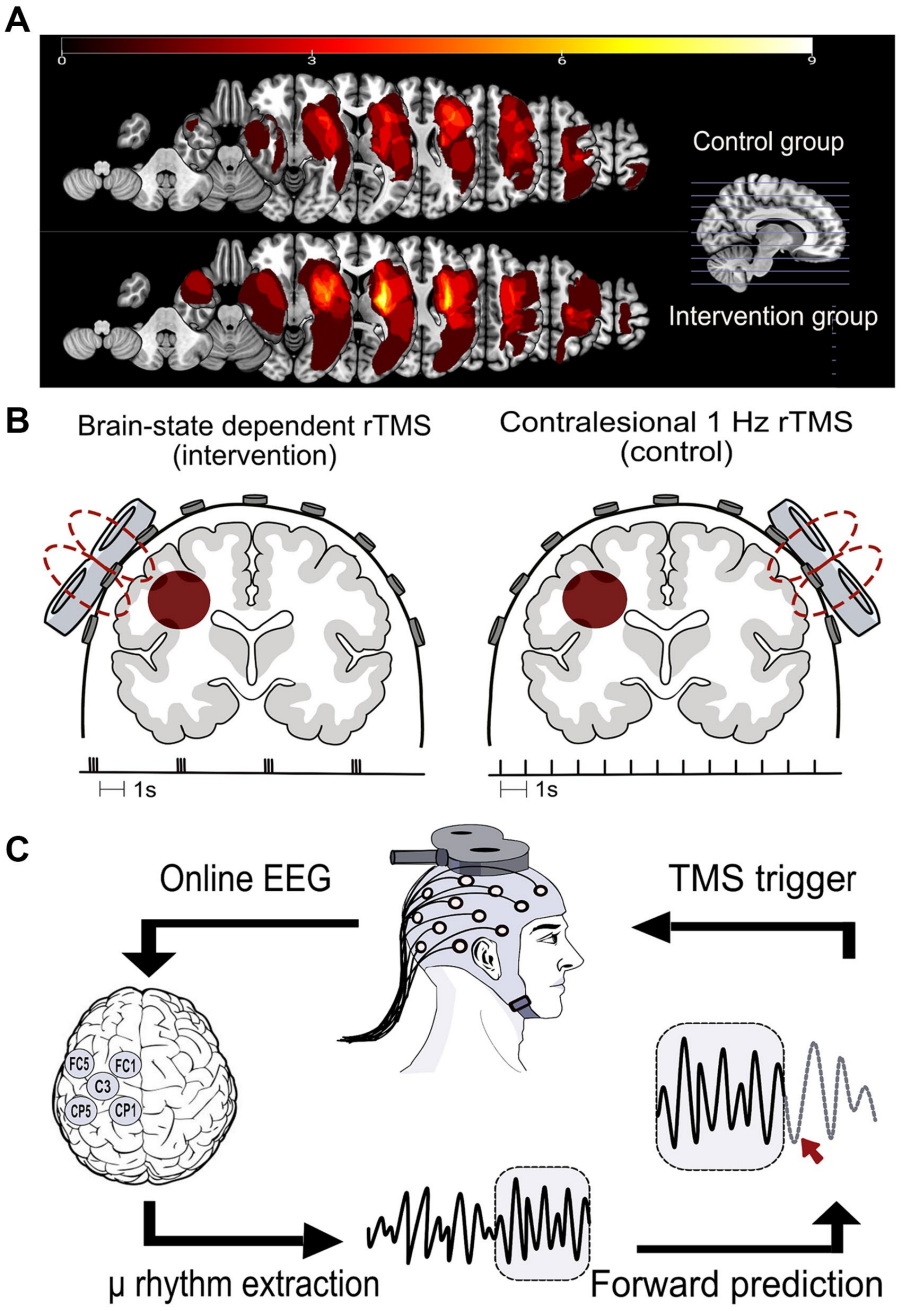
**(A)** Lesion distribution overlays for both study groups, the control group (upper panel, *n* = 11) and the intervention group (lower panel, *n* = 13), were generated through MRI analysis. The computation involved manual delineation conducted by a qualified neurologist for each patient to define the volume of interest (VOI) within the subject space. Subsequently, the VOI was normalized to Montreal Neurological Institute (MNI) space using the Clinical Toolbox in SPM12. VOIs were mirrored to the left in right hemisphere lesions. Lesion overlap was calculated utilizing the NiiStat Toolbox functions for MATLAB. **(B)** The two rTMS protocols used in this study. Left: the brain state-dependent stimulation group (intervention group) received 1,200 TMS pulses to the ipsilesional M1 in the form of triple pulses at 100 Hz and an interstimulus interval of on average 3 s. Right: the control group received 1,200 TMS pulses to the contralesional M1 at 1 Hz frequency. **(C)** The brain state-dependent stimulation method: an output copy of the EEG signal was analyzed in real-time in order to determine the timing of and trigger the TMS pulse. The local cortical activity of the sensorimotor cortex μ-rhythm was extracted using a surface Laplacian source derivation montage centered around C3 for a left-hemisphere lesion. A sliding 500 ms window of EEG data was band-pass filtered (6–15 Hz) to isolate the μ-rhythm. An autoregressive model was used to predict the instantaneous phase of the signal. When the band-power exceeded a predetermined threshold and simultaneously matched a pre-specified phase, a TMS pulse was triggered.

Additionally, magnetic resonance images (MRIs) (clinical or research scans, T1 or T2-weighted) were available for a subset of patients (*n* = 24; 11 in the control group and 13 in the intervention group), and used for lesion mapping. Lesions were overlaid for each group (Figure 2A), guiding their classification as subcortical, cortical, or combined, with motor cortex involvement defining cortical lesions (Table 1).

#### 2.3.1 TMS stimulation protocols

Both groups received 12 rTMS therapy sessions over 4 weeks (3 sessions per week). During rTMS stimulation sessions, patients were seated on an electronically adjustable reclining chair (Treatment Chair with Neckrest, MagVenture, Farum, Denmark) with both arms relaxed. A conventional TMS stimulator (MagPro R30, MagVenture, Farum, Denmark) was used to deliver biphasic TMS pulses to the motor hotspot, using an air-cooled figure of eight coil (MCF-B65 MagVenture, diameter 75 mm) according to the designated protocol. The coil was oriented such that the second phase of the biphasic pulse induced an electrical field in the brain from lateral-posterior to medial-anterior.

In the 1 Hz rTMS group (control group), 1,200 TMS pulses were delivered at a frequency of 1 Hz and an intensity equivalent to 115% RMT to the motor hotspot of the contralesional M1 (8). For the brain state-dependent stimulation group (intervention group), phase-specific TMS pulses were triggered by a customized real-time signal-processing system (23). Triple pulses at 100 Hz, with an inter-burst interval of on average 3 s were delivered based on the instantaneous oscillatory phase of the ipsilesional sensorimotor μ-rhythm (28). A total of 400 triplets at 100% RMT were triggered using a combined criterion of μ-phase (triggered at negative peak) and power threshold. The power threshold was individually adjusted to result in a stimulation frequency around 0.33 Hz (Figure 2B). The intervention protocol builds on previous theta-burst inspired paradigms used in our group, which reliably induced plasticity in healthy participants (23, 28). A stimulation intensity of 100% was used to reliably elicit MEPs using triple bursts, providing an additional measure for coil position stability during therapy.

Immediately following each rTMS session, patients engaged in a 50 min personalized exercise-based physiotherapy training targeting arm and hand function. The training involved practicing meaningful functional tasks tailored to the patients’ goals and motor abilities, covering various aspects like trunk control, strength training, object manipulation, and fine motor training. In total, patients received 600 min (12 sessions × 50 min) of physiotherapy throughout the study period.

### 2.4 EEG and EMG recordings

NeurOne Tesla biosignal amplifier (Bittium Biosignals Ltd., Finland) was used for acquiring both EEG and EMG signals. Scalp EEG was recorded using a TMS-compatible 64-channel Ag/AgCl sintered ring electrode cap (EasyCap GmbH, Germany) arranged according to the International 10-5 system with a denser electrode array over the sensorimotor cortex (39). The reference electrode was located at the FCz position, and the ground electrode was located at the POz position. During rTMS therapeutic stimulation sessions, the EEG caps placed on the patients’ heads were equipped with only 5 electrodes in the C3-centered or C4-centered Laplacian montage (see Figure 2C), along with reference and ground electrodes. EEG electrodes were fitted onto the EEG cap on the stimulated side in both therapy groups.

EEG signal was sampled at a rate of 5 kHz and low pass filtered (1.25 kHz cut-off). EMG signal was sampled at 10 kHz and low pass filtered (1.5 kHz cut-off) using bipolar surface electrodes (Ambu^®^ Neuroline 720, Denmark) placed on the abductor pollicis brevis (APB), first dorsal interosseous (FDI) and abductor digiti minimi (ADM) in a belly-tendon arrangement. An additional ground electrode was placed on the styloid process of the ulna.

Resting-state EEG data (5 min) was acquired during the pre-assessment, as well as during post-treatment, and follow-up sessions. Before the recording, the skin was carefully prepared by mild skin abrasion (Nuprep Skin Prep Gel, United States). To ensure signal quality, care was taken to maintain electrode impedances below 10 kΩ. Patients were instructed to keep their eyes open, and to maintain their neck, jaw, and arm muscles relaxed during the recording. The experimenter continuously monitored the EMG and EEG signals during the recording, and took appropriate measures whenever artifacts were introduced.

### 2.5 Motor hotspot identification and resting motor threshold measurement

The stimulation target was the hand knob area in M1. For each participant, the motor hotspot was determined by identifying the coil position and orientation over the M1 hand representation that elicited the largest and most consistent MEPs in either ABP, FDI or ADM muscles. The identified muscle—in the affected upper limb for the intervention group or the unaffected upper limb for the control group—was designated as the reference muscle for each participant, and used consistently throughout all subsequent therapeutic sessions.

To ensure the most effective hand knob stimulation, a hotspot search was performed at the beginning of each stimulation session to re-identify the reference muscle’s hotspot. Once identified, the motor hotspot was marked on the EEG cap to guarantee consistent coil positioning throughout the session.

To determine the stimulation intensity, we assessed the RMT in the reference muscle every session. RMT was defined as the minimum stimulus intensity required to elicit MEPs with a peak-to-peak amplitude of at least 50 μV in the target muscle (the muscle with the lowest RMT) in at least 5 out of 10 consecutive TMS pulses delivered to the motor hotspot (40).

### 2.6 Real time stimulation method

EEG-phase synchronization was achieved using a real-time EEG data analysis method described in Zrenner et al. (23). Briefly, an algorithm implemented in Simulink Real-Time (Mathworks Ltd., United States, R2017b) was used to analyze an online output copy of the EEG signal in real-time in order to determine the timing of and trigger the TMS pulse. The local cortical activity of the sensorimotor cortex μ-rhythm was extracted using a surface Laplacian source derivation montage centered at electrode C3 for left-sided lesions (referenced to the average of the surrounding electrodes CP1, CP5, FC1, and FC5), and C4 for right-sided lesions (referenced to CP2, CP6, FC2, and FC6) (21–23, 41). A sliding window (length 500 ms) of data was band-pass filtered (6–15 Hz) to isolate the μ rhythm. This relatively wide range was chosen as individual peak frequencies of the ipsilesional sensorimotor cortex spanned a wider range than the typical alpha band. The instantaneous phase at the time of the TMS trigger decision was estimated by forward-predicting the signal using an autoregressive model. The spectral band-power within the sliding window of data was also estimated in real-time using fast Fourier analysis (FFT). When the band-power exceeded a predetermined threshold and simultaneously matched a pre-specified phase, a TMS pulse was triggered (Figure 2C). The power threshold was adjusted continuously during the experiment to achieve an average stimulation frequency of 0.33 Hz.

#### 2.6.1 Spectral estimation

Spectral estimation was performed on the resting state EEG data obtained during screening sessions using the multi-taper method, applying three distinct tapers (window functions) to baseline-corrected epochs with a length of 1.4 s and a 50% overlap. The aperiodic fractal background component of the spectrum was estimated using the irregular-resampling auto-spectral analysis (IRASA) method (42) with factors 1.1 to 2.9 in steps of 0.1 and excluding 2.0, and removed from the full spectrum.

The resulting ratio between the power of the periodic component and the aperiodic background noise can be termed signal-to-noise ratio (SNR). SNR determines the maximum achievable precision of phase targeting (43). The individual μ-peak frequency was determined from the corrected spectrum in the range between 6 and 15 Hz of both the ipsilesional and contralesional hemispheres, and the respective SNR at that frequency was obtained.

#### 2.6.2 Real-time EEG phase estimation accuracy

Phase estimation accuracy for each subject was calculated using the resting state EEG dataset. We compared the real-time—causal—phase estimation, where only data preceding the time of interest, i.e., the TMS pulse is available, against a “benchmark” estimation method using the PHASTIMATE toolbox (43). The benchmark—non-causal—estimation uses data before and after the time point of interest for instantaneous phase and amplitude determination.

Both (simulated) real-time phase estimate and the non-causal benchmark phase were obtained from TMS-artifact-free resting-state EEG data. For details, please refer to Zrenner et al. (44). Briefly, resting-state EEG data was segmented into overlapping 1.4 s epochs. The phase at the center of each epoch was estimated using both methods. The causal real-time algorithm utilized a 500 ms segment of data preceding the center of the epoch, while the non-causal benchmark method utilized the entire epoch for estimation. Epochs with an alpha oscillation spectral power below the median were discarded from the phase accuracy analysis, to simulate the effect of the real-time amplitude threshold. We then computed the average error between the trial-wise causal and benchmark estimates. Circular standard deviation quantified the variance of trial-wise errors, providing a measure of estimation precision.

### 2.7 Spasticity-related measurements

#### 2.7.1 Objective assessment of spasticity in the wrist flexors using a hand-held dynamometer

The Portable Spasticity Assessment Device (PSAD) from Movotec, Denmark, a hand-held dynamometer, was utilized for objective wrist flexor spasticity assessment (45, 46). It enabled simultaneous measurement of force, joint movement, and muscle activity to precisely measure reflex-mediated muscle resistance (spasticity) and distinguish it from soft tissue (passive) stiffness.

Passive stiffness was evaluated by slow wrist joint extension (<20°/s) to assess resistance in the absence of a stretch reflex, while reflex-mediated stiffness was measured during high-velocity joint movement (>300°/s) triggering a stretch reflex. Six repetitions were performed for each velocity. EMG data was recorded using bipolar surface adhesive electrodes placed over the bellies of both flexor carpi radialis (FCR) and extensor carpi radialis (ECR) muscles. The PSAD was wirelessly connected to a data acquisition software providing real-time visual feedback on stretch velocity and EMG activity. All data, including position, acceleration, angular velocity, EMG, and forces, were saved for offline analysis. A MATLAB code was used to analyze the raw data and extract the outcome parameters (passive stiffness and stretch reflex torque) based on the methods detailed in Mahmoud et al. (45).

#### 2.7.2 Electrophysiological assessment of post-activation depression

Post-activation depression, a spinal inhibitory mechanism characterized by frequency-dependent reduction in neurotransmitter release, is consistently decreased on the affected upper limb in spastic stroke patients (47, 48). This reduction correlates with clinical spasticity assessments (48), potentially contributing to stretch reflex hyperexcitability. To investigate potential spasticity-related neurophysiological changes induced by rTMS, we measured post-activation depression by varying the inter-stimulus interval (ISI) of consecutive stimuli to the median nerve, while measuring the H-reflex in the FCR (47).

Using a direct current stimulator (DS07 Digitimer, United Kingdom), the median nerve was stimulated at the elbow with 1 ms rectangular shocks via metal electrodes (brass, 1.5 cm radius). Stimulation intensity was adjusted to produce an H-reflex with a peak-to-peak amplitude of Hmax/2. Stimulation blocks alternated ISIs of 8 or 2 s until 30 reflexes at each interval were obtained (3 blocks of 10 stimuli per ISI). EMG data from NeurOne was analyzed using a customized Python program. The peak-to-peak amplitudes of the individual H-reflexes were extracted, and the responses triggered with the same ISI were averaged and saved for statistical analysis. The outcome measure of this assessment is the calculated ratio of the H-reflex amplitude evoked every 2 s to that of the H reflex evoked every 8 s: (H2/H8).

### 2.8 Clinical outcome measures

The primary outcome measure was defined as the change in motor impairment measured using the standardized FMA-UE (49) both immediately post-treatment (post), and 3 months post-treatment (follow-up) in comparison to baseline.

Other outcome measures included the Wolf Motor Function Test (WMFT), which comprised 15 timed tasks for the evaluation of upper limb motor function (50). Both the time (in seconds) required to perform a task, as well as the quality of the movement were recorded. Movement quality was rated on a scale from 0 to 5. Total time score was calculated by summing the time taken for each task (capped at 120 s if not completed within that time frame), while the quality score is the sum of all task quality ratings, with a maximum score of 75.

Clinical measures of spasticity were obtained using the modified Ashworth scale (MAS) (51), where the examiner passively mobilized the joint of interest and simultaneously estimated the perceived resistance according to a 6-point ordinal scale. The total MAS score was calculated by summing individual scores from 14 movements of multiple upper limb joints (flexion, abduction, internal rotation and external rotation of the shoulder; flexion and extension of the elbow; pronation and supination of the forearm; flexion and extension of the wrist; flexion and extension of the fingers; and adduction and abduction of the thumb).

Patient-centered outcome measures included the visual analogue scale (VAS) for self-reported spasticity, where patients reported the degree of spasticity they perceived in their arm on a scale that ranged between 0 and 100. The Motor Activity Log (MAL-30) a questionnaire where patients described the frequency with which they used the affected limb to perform each of 30 different activities of daily living (52, 53). Additionally, the Disability Rating Scale (54) was used for assessing the impact of spasticity on daily activities.

### 2.9 Statistical analysis

Statistical analyses were performed on the intention-to-treat (ITT) population using IBM-SPSS Statistics software version 28.0.1.1 (IBM Corp., Armonk, NY, United States), JASP version 0.18.0 (55) was used for Bayesian statistics. Baseline comparability between the intervention and control groups included the examination of several variables, namely age, time since stroke, and baseline FMA-UE scores using an independent *t*-test.

To assess normality assumptions, we inspected histograms for each outcome measure and calculated skewness and kurtosis, we also ran the Shapiro–Wilk test for each assessment session and each group. The assumption of sphericity was tested using Mauchly’s test, which indicated no violation of sphericity for any variable, eliminating the need for correction. For normally distributed datasets, we employed repeated measures ANOVA, considering Session (baseline, post, follow-up) as a within-subject effect, and Group (Intervention, Control) as a between-subject effect. Additionally, we examined the interaction (Session × Group). The Bayesian repeated measures ANOVA used BF_10_ and compared to null model.

When data did not satisfy the assumption of normality (WMFT-time, WMFT-function and stretch reflex torque), We used Generalized Estimating Equations (GEE) to test the effect of Session, Group and Session × Group interaction. We applied a GEE model with an exchangeable correlation structure to account for within-subject correlations over time. The outcome variable was treated as continuous, with a gamma probability distribution and a log link function. Robust estimation was used to handle violations of normality. A *p*-value of <0.05 was considered to indicate statistical significance. For ordinal data (Disability Rating Scale), we used Whitney U test.

## 3 Results

Seventy-nine subjects were screened between October 2019 and March 2022. Thirty stroke patients (23 males, 7 females), average age 57.9 ± 8.0 years, participated in this study. All patients had unilateral hemiparesis due to stroke at least 6 months before participation (average 51 ± 47 months). A participant flow diagram is presented in Figure 1. The characteristics of the study participants are presented in Table 1. Two patients from the control group were lost to follow-up and one additional patient refused to perform the WMFT during follow-up.

### 3.1 Adverse events

During the study, adverse events were observed in 7 out of 360 therapy sessions: 4 in the control group and 3 in the intervention group. In the control group, adverse events included tiredness (*n* = 1), headache (*n* = 1), and shoulder/arm pain—presumably, posture, not rTMS-related—(*n* = 2, 1 session terminated). In the intervention group, adverse events consisted of head numbness (*n* = 1), transient double vision (*n* = 1), and tiredness (*n* = 1). Two pre-assessment sessions had to be terminated—one due to orthostatic syncope and another due to intense agitation during TMS.

### 3.2 Spectral analysis and phase estimation accuracy

The mean μ-peak frequency of the ipsilesional sensorimotor cortex was 9.5 ± 2.7 Hz with a mean SNR of 5.4 ± 2.3 dB, compared to 12.5 ± 2.3 Hz and 4.9 ± 2.3 dB in the contralesional sensorimotor cortex. With regards to targeting the μ-oscillation in the ipsilesional hemisphere, the average phase difference between the estimate of the real-time algorithm and the post-hoc phase measure was 1° ± 83.6° (mean ± circular standard deviation) demonstrating an absence of a systematic bias. These results are similar to findings reported in the literature (31, 44).

### 3.3 Group comparison

#### 3.3.1 Baseline group comparison

The results of the *t*-test revealed no significant differences between the two groups at baseline with regards to age (intervention: 57 ± 8 years, control: 60 ± 9 years; *p* = 0.47), time since stroke (intervention: 47 ± 52 months, control: 54 ± 44 months; *p* = 0.41), and FMA-UE scores (intervention: 37.6 ± 15, control: 38 ± 15; *p* = 0.9).

#### 3.3.2 Effects on clinical outcomes

**Figure 3:**
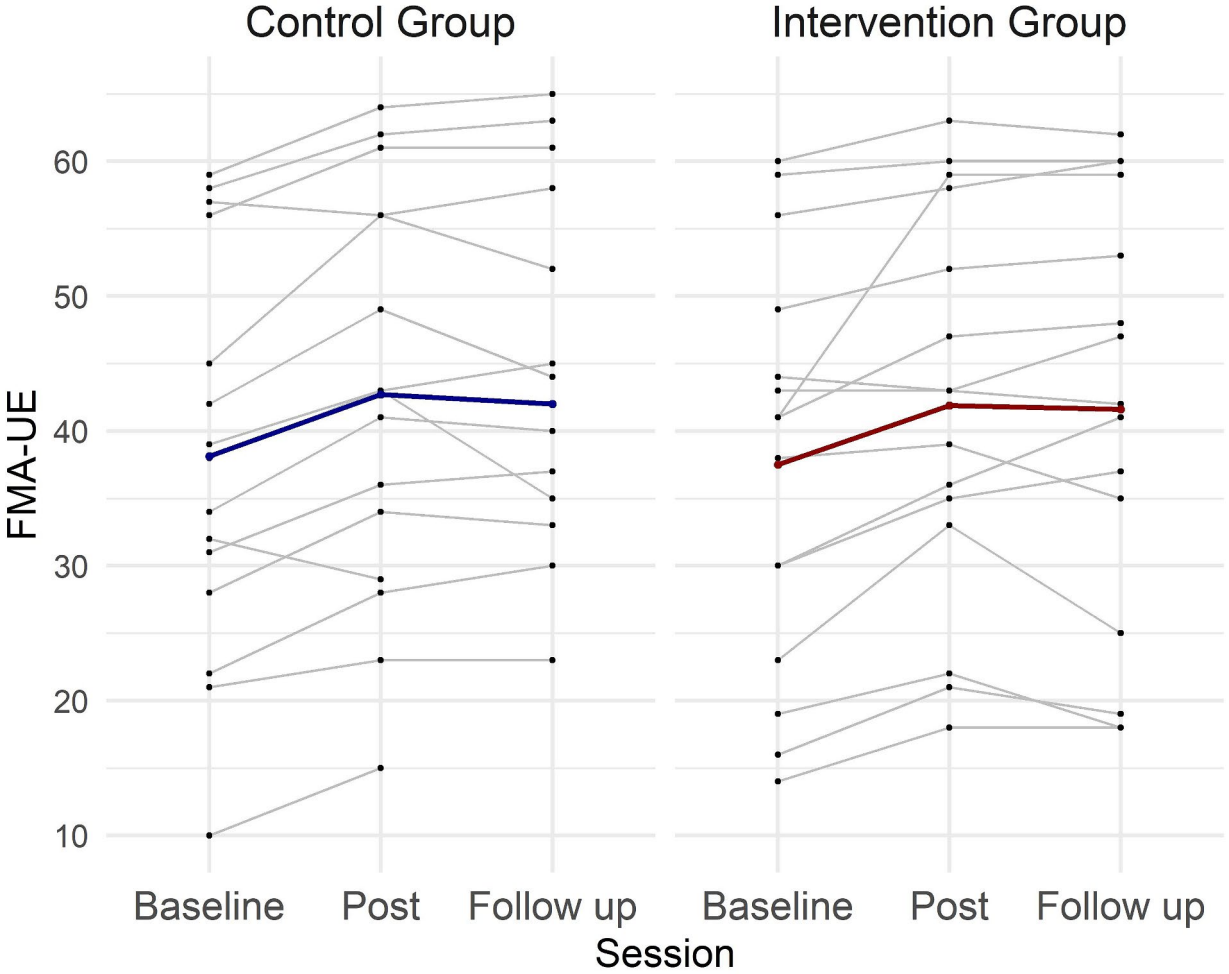
Individual and group FMA-UE scores across sessions for the intervention and control groups. Each thin line represents one patient, while the thick lines represent the mean values for each group across the three evaluation sessions.

The repeated measures ANOVA (Table 2) revealed a significant effect of the treatment on FMA-UE score [Session: *F*_(2,52)_ = 25.7, *p* < 0.001]. No difference was observed between the groups [Group: *F*_(2,26)_ = 0.45, *p* = 0.51], and there was no significant Session × Group interaction [*F*_(2,52)_ = 0.31; *p* = 0.73]. *Post hoc* analyses indicated sustained gains in FMA-UE at 3 months follow up, with a significant difference between baseline and post scores (*p* < 0.001), baseline and follow-up scores (*p* < 0.001), but not between post and follow-up scores (*p* = 0.48) (Figure 3).

**Table 2:** Results of the statistical analysis for clinical outcome measures.

	Contralesional 1 Hz rTMS (Control) mean ± standard deviation			Phase-dependent rTMS (Intervention) mean ± standard deviation					
Measure	Pre	Post	Follow-up	Pre	Post	Follow-up	Session	Group	Session × Group
FMA-UE/66	40.8^a^ ± 3.9	45.8 ± 4.0	45.1 ± 4.1	37.6 ± 3.7	41.6 ± 3.7	681 ± 121	*F*_(2,52)_ = 25.7, *p* < 0.001	*F*_(2,26)_ = 0.45, *p* = 0.51	*F*_(2,52)_ = 0.31, *p* = 0.73
WMFT-time (s)	652 ± 135	518 ± 126	564 ± 132	731 ± 125	659 ± 116	40.2 ± 5.6	*χ*^2^ (2) = 22.5, *p* < 0.001	*χ*^2^ (1) = 0.13, *p* = 0.72	*χ*^2^ (2) = 3.5, *p* = 0.18
WMFT-function/75	42.8 ± 5.7	46.8 ± 5.7	46.7 ± 6	37.9 ± 5.2	40.2 ± 5.2	8.5 ± 1.9	*χ*^2^ (2) = 26.2, *p* < 0.001	*χ*^2^ (1) = 0.04, *p* = 0.85	*χ*^2^ (2) = 4.1, *p* = 0.12
Grip strength (kg)	15.3 ± 2.1	15.6 ± 2.4	15.8 ± 2.0	9.0 ± 2.0	9.4 ± 2.2	0.92 ± 0.33	*F*_(2,44)_ = 0.09, *p* = 0.91	*F*_(2,22)_ = 5.7, *p* = 0.03	*F*_(2,44)_ = 0.21, *p* = 0.81
Stretch reflex torque (mV)	1.53 ± 0.34	1.29 ± 0.28	1.58 ± 0.33	1.29 ± 0.34	0.93 ± 0.28	7.5 ± 1.3	*χ*^2^ (2) = 5.9, *p* = 0.05	*χ*^2^ (1) = 1.2, *p* = 0.27	*χ*^2^ (2) = 2.1, *p* = 0.36
MAS_total /70	9.8 ± 1.3	8.2 ± 1.3	8.1 ± 1.4	8.3 ± 1.2	6.6 ± 1.2	33.8 ± 5.8	*F*_(2,50)_ = 5.8, *p* = 0.005	*F*_(2,25)_ = 0.48, *p* = 0.49	*F*_(2,50)_ = 0.51, *p* = 0.60
MAL (%)	23.9 ± 5.6	36.9 ± 7	31.6 ± 6.6	24.9 ± 4.9	31.0 ± 6	33.8 ± 5.8	*F*_(2,42)_ = 11.6, *p* = <0.001	*F*_(2,21)_ = 0.01, *p* = 0.92	*F*_(2,42)_ = 2.1, *p* = 0.13

FMA-UE, Fugl-Meyer Assessment-Upper Extremity; WMFT, Wolf Motor Function Test; MAS, modified Ashworth scale; MAL, Motor Activity Log.

a The discrepancy in group mean values between the baseline group comparison analysis and this table is due to the loss of two patients to follow-up. ANOVA systematically excludes individuals with missing data, resulting in means calculated considering only subjects with a complete dataset.

Potential FMA-UE treatment effects were further explored using a Bayesian repeated measures ANOVA. The analysis revealed very strong evidence for an effect of Session (BF_incl_ = 616569.643), but showed evidence against Group (BF_incl_ = 0.508) and Session × Group interaction (BF_incl_ = 0.907). *Post hoc* comparison for Session demonstrated very strong evidence for a difference between baseline and post (BF_10,U_ = 23733.611), baseline and follow-up (BF_10,U_ = 1055.822), but evidence against a difference between post and follow-up (BF_10,U_ = 0.246). These results align with the findings of the classical statistical analysis.

The WMFT showed a significant treatment effect (Session) for both time [*χ*^2^ (2) = 22.5, *p* < 0.001] and function components [*χ*^2^ (2) = 26.2, *p* < 0.001]. No Group effect or significant Session × Group interactions were observed. Moreover, there was a significant effect of the treatment on the reduction of stretch reflex torque measured with the hand-held dynamometer [Session: *χ*^2^ (2) = 5.9, *p* = 0.05], without a significant Group effect or Session × Group interaction.

**Figure 4:**
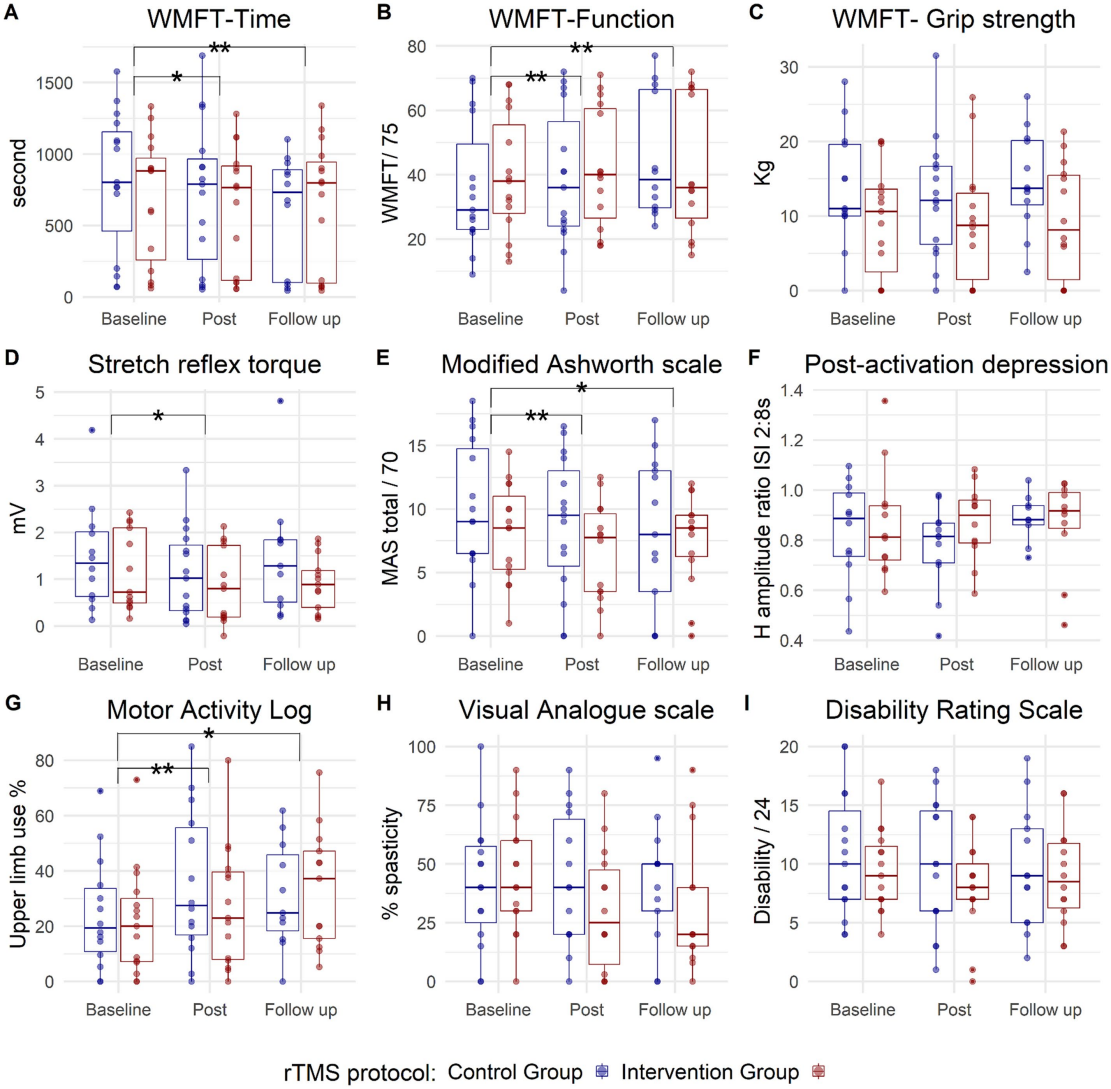
Box plots depicting group data for the two study groups: contralesional 1 Hz rTMS (control) group in blue and phase-dependent (intervention) group in red, across three measurement points (baseline, post, and follow-up). Each panel corresponds to one outcome measure **(A)**: WMFT-Time, **(B)**: WMFT-Function, **(C)**: WMFT-Grip strength, **(D)**: Stretch reflex torque, **(E)**: Modified Ashworth scale, **(F)**: Post-activation depression, **(G)**: Motor Activity Log, **(H)**: Visual Analogue scale, **(I)**: Disability Rating scale. The small blue and red circles represent individual patient data. The horizontal line inside the box represents the median value. The box expands between the 25th percentile (Q1) to 75th percentile (Q3), while the whiskers extend to the smallest and largest data points within 1.5 of the interquartile range (Q3 to Q1). Points outside this range could be considered outliers. WMFT, Wolf Motor Function Test. The asterisk represents a significant effect of the treatment (Session) on both groups, with no Session × Group interaction, (*) is significance at *p* < 0.05 level, while (**) represents significance at *p* < 0.001 level.

The MAS (total) exhibited a significant decrease after treatment [Session: *F*_(2,50)_ = 5.8, *p* = 0.005], without a significant Session × Group interaction. Patient-reported use of the upper extremity in everyday activities (MAL) significantly increased after treatment [Session: *F*_(2,42)_ = 11.6, *p* < 0.001] without a difference between the groups. No significant effects of the treatment were observed on grip strength, post-activation depression, VAS for perceived spasticity, or Disability Rating Scale (Figure 4).

## 4 Discussion

Evidence from experiments in healthy subjects indicates that brain state-dependent rTMS may improve the magnitude and consistency of rTMS effects by delivering the stimulation during periods of increased cortical excitability, thus, increasing the induction of LTP-like plasticity (23, 26). In this trial, we investigated feasibility and efficacy of an rTMS protocol which synchronized the TMS bursts with the trough of the ipsilesional sensorimotor cortex μ-rhythm, i.e., a high-excitability state of the corticospinal tract. We compared this intervention to standard low-frequency rTMS of the contralesional motor cortex.

The stimulation was well-tolerated in both groups. All adverse events were mild, mostly standard TMS side-effects or unspecific in nature. The accuracy of targeting the trough of the ipsilesional sensorimotor cortex μ-rhythm was comparable to previous studies in healthy subjects (23, 27, 44), confirming the feasibility of safe and successful administration of brain state-dependent stimulation to chronic stroke patients in a clinical setting. A more detailed analysis of EEG parameters and changes will be published in a separate manuscript.

The analysis of clinical endpoints demonstrated significant improvements in motor impairment and function, alongside a reduction in clinical and objective measures of spasticity in both groups with no significant difference between the groups. Motor improvement was maintained 3 months’ post-treatment, and was accompanied by an increased use of the affected upper limb in daily activities, as indicated by the MAL questionnaire. Although no significant difference was observed between the two groups, this finding does not necessarily mean that the stimulation protocols have identical effects. The study’s design, with its limited statistical power, may not have been sensitive enough to detect differences, if present. Therefore, while this feasibility study was not intended to compare the effectiveness of the stimulation protocols, it serves as a pilot to inform the design of larger clinical trials.

In addition, the absence of a sham-rTMS arm in this study raises the possibility that clinical improvements in both groups may be attributed to the physiotherapy, especially considering the limited physiotherapy typically received by chronic patients. Our study did not aim to establish rTMS superiority over physiotherapy, but rather to compare two rTMS protocols that serve as priming tools for enhancing motor rehabilitation. However, numerous randomized sham-controlled trials have demonstrated the efficacy of rTMS interventions in improving motor impairment and function in chronic stroke. For comprehensive reviews of these trials, please refer to Graef et al. (2), Kim et al. (56), Lüdemann-Podubecká et al. (57), Dionisio et al. (58), and Zhang et al. (59).

Both rTMS protocols implemented in this study aimed to increase the excitability of the ipsilesional hemisphere and are embedded in the interhemispheric inhibition (IHI) model. The model supposes that unopposed inhibition from the contralesional to the ipsilesional hemisphere impedes recovery after stroke. The phase-dependent stimulation aimed to directly upregulate the excitability of the ipsilesional cortex using high-frequency (100 Hz) triplets, while the low-frequency (1 Hz) rTMS attempted to indirectly upregulate the excitability of the ipsilesional cortex through downregulation of the contralesional hemisphere and thereby rebalancing abnormal interhemispheric inhibition. The integrity of the ipsilesional corticospinal tracts has been proposed as a biomarker for potential benefit from therapies that aim to upregulate ipsilesional excitability (60). Despite the different working mechanisms of the two stimulation protocols used in this study, both aimed to enhance the excitability of the ipsilesional hemisphere. In line with this, only patients with positive ipsilesional MEPs were included, thereby increasing both the homogeneity of the study sample and the potential for improvement.

Selection of contralesional low-frequency stimulation as the control intervention was based on the cumulative evidence supporting its efficacy. However, recent literature questions its mechanism and suitability for all stroke patients (61). Indeed, the IHI imbalance model itself is currently being debated and re-evaluated in light of recent evidence (62–66), suggesting that IHI *per se* should not be a target for rTMS interventions (65). Contemporary models of interhemispheric communication present a more complex picture of the interaction between the two hemispheres following stroke, contingent on factors such as structural reserve (67) and integrity of the callosal and frontal connections (68). It is crucial to emphasize, however, that the ongoing debate around the IHI model does not invalidate previously reported beneficial effects [for an overview, see: Kim et al. (56) and Starosta et al. (69)]. Rather, it suggests that these effects could arise from different, yet unknown mechanisms (65).

Two key principles are proposed to enhance the effectiveness of therapeutic brain stimulation: precision and personalization (70). This proof-of-concept study showed that the brain state-dependent stimulation protocol produced outcomes comparable to an established conventional open-loop stimulation protocol. This makes synchronization of brain stimulation to individual brain oscillations a promising possibility for personalizing therapeutic rTMS interventions. It is necessary to acknowledge, however, that this trial is preliminary. It was designed as a proof-of-concept trial with limited statistical power and a single control group which precludes further in-depth analyses or comparisons. Also, the study does not offer sufficient evidence to support the application of EEG brain-state dependent rTMS in standard clinical neurorehabilitation settings. Comprehensive investigations are necessary to explore the full potential of this technology. Currently, a confirmatory multicenter randomized controlled trial is ongoing in Germany to test the therapeutic efficacy in subacute motor stroke patients (ClinicalTrials.gov, identifier: NCT05600374) (71).

A primary strategy to improve the effectiveness of this approach is through patient selection and stratification. The selection of an ideal rTMS protocol should consider factors like stroke type, location, and the stage of stroke recovery. In addition, the future of therapeutic brain stimulation should encompass enhanced subject stratification through in-depth analysis of brain connectivity and structural integrity. In the context of optimizing phase-dependent stimulation protocols specifically, several considerations arise. Firstly, it may be necessary to choose patients with a sufficiently high μ-rhythm power and SNR for optimal phase targeting (43). Secondly, given heterogeneity in lesion location and size among the stroke population, EEG channel selection may be essential. Thirdly, parameters for brain state-dependent stimulation could be entirely automated using personalized classifiers (72, 73).

### 4.1 Effects on spasticity

When evaluating the impact of the rTMS therapy on spasticity, our investigation revealed a significant reduction in both MAS-total and the MAS-wrist extension. These results align with previous studies (74–77). This study represents one of the first attempts to objectively assess the impact of rTMS on spasticity. Using recently developed technology, we found a significant reduction in the stretch reflex torque, which represents the neurogenic component of resistance to joint stretch. No change was recorded in the passive stiffness components. These findings are similar to what we observed in a previous study (46) which exclusively involved patients receiving 1 Hz contralesional rTMS.

Here, we found that high-frequency rTMS of the ipsilesional M1 (brain state-dependent) also reduced spasticity. The finding that both stimulation protocols were similarly able to improve motor function and to reduce spasticity may indicate that reduction of spasticity could be related to motor function improvement, a finding that we recorded previously (46). This improvement, which may be reflected in an increased excitability of the ipsilesional corticospinal tract can trigger changes at the level of the spinal cord and possibly the motor neurons itself, which lead to a reduction in the stretch reflex-mediated torque. We also examined the possibility of the involvement of the post activation depression as a putative mechanism underlying the measured reduction in spasticity. This was not confirmed from this data, in line with our previous study, which examined this effect only by applying the 1 Hz rTMS protocol to contralesional M1 (46).

Recent evidence suggests that motor impairment and the development of spasticity share a common pathophysiological mechanism: the upregulation of descending motor tracts originating in the brainstem, primarily the reticulospinal tract (78, 79). This upregulation emerges as a response to the reduced descending input to the spinal motoneurons following an upper motor neuron lesion, thereby supporting muscle activation despite the loss of corticospinal drive. Additionally, the lesion itself may reduce cortical activation of the inhibitory brainstem centers by affecting the corticoreticular tract. These changes can enable the production of force in otherwise weak muscles, but also alter the excitability of various spinal circuits, ultimately increasing the excitability of the stretch reflex, leading to spasticity (78).

The finding that both stimulation protocols examined in this study improve motor function and reduce spasticity to a similar extent (pending confirmation by larger trials) could suggest that these protocols influence the same underlying mechanism, though in different ways. For instance, ipsilesional high-frequency stimulation might enhance corticospinal tract excitability, and increase inhibition of the brainstem centers by boosting corticoreticular tract excitability, which would modulate reticulospinal tract input. Conversely, contralesional inhibitory stimulation could inhibit ipsilateral motor cortical areas (M1, somatosensory areas and premotor cortex) that facilitate the brainstem descending tracts. Understanding these physiological mechanisms requires an in-depth examination of the excitability of different cortical areas and descending tracts. While speculative due to the lack of evidence from this trial, exploring these mechanisms could be of great value for understanding and optimization of rTMS intervention protocols.

## 5 Conclusion

This study demonstrates that brain state-dependent stimulation can be successfully applied in chronic stroke patients with clinical effects that are similar to current TMS standard therapy. EEG-TMS therefore constitutes an exciting option for personalization and optimization of motor neurorehabilitation supported by non-invasive brain stimulation. Future studies should further explore its clinical benefits and optimize its application.

## Data Availability

The raw data supporting the conclusions of this article will be made available by the authors, without undue reservation.
